# Effect of ceramic material type on the fracture load of inlay-retained and full-coverage fixed dental prostheses

**DOI:** 10.1080/26415275.2020.1744443

**Published:** 2020-03-30

**Authors:** Hamid Kermanshah, Fariba Motevasselian, Saeedeh Alavi Kakhaki, Mutlu Özcan

**Affiliations:** a Restorative Dentistry department, Tehran University of Medical Sciences School of Dentistry, Tehran, Iran; b Clinic for Fixed and Removable Prosthodontics and Dental Materials Science, University of Zurich, Zurich, Switzerland

**Keywords:** Fracture load, monolithic zirconia, zirconia reinforced lithium silicate

## Abstract

**Objective:**

Ceramic inlay-retained fixed partial denture (IRFPD) is a conservative prosthetic option but the mechanical durability of new high strength zirconia reinforced glass ceramic FPDs is not investigated. The purpose of this study was to compare fracture load of 3-unit ceramic FPDs.

**Materials and methods:**

Extracted premolars and molars (*N* = 64) were used to create three test groups (IRFPDs) and one control group (full coverage FPD) (*n* = 8). The teeth were embedded in PMMA resin with a mesiodistal distance of 6 mm. Premolars had a distal and molars had a mesial inlay preparation (width: 3 mm; height: 4 mm) in the test groups. IRFPDs were made from a zirconia reinforced lithium silicate (VS) or a monolithic zirconia. Zirconia IRFPDs received 2 types of surface treatments: sandblasting (Zr-IRFPD) or internal coating with feldspathic porcelain (ZrC-IRFPD). Control group was made from monolithic zirconia with the same connector size and zirconia surfaces were sandblasted (Zr-FPD). All restorations were cemented using a resin luting cement. After 5000 thermo-cycles, fracture load values (N) were determined with a universal testing machine at a crosshead speed of 0.75 mm/min. Data were analyzed using 1-way ANOVA and Tukey`s post hoc test (*p ˂* .05).

**Result:**

Fracture load (mean ± SD) of Zr-FPDs, Zr-IRFPDs and ZrC-IRFPDs were 672 ± 183, 672 ± 123 and 638 ± 59, respectively, being not statistically different (*p* > .05). VS-IRFPD exhibited statically lower values (391 ± 136). The predominant mode of failure was fracture at the connector area in all groups.

**Conclusion:**

The fracture load of 3-unit IRFPD was significantly affected by types of ceramics but the retainer design and surface treatment in Zr groups did not show a significant effect.

## Introduction

Replacement of missing teeth could be achieved with numerous artificial materials. Conventional full-coverage tooth-supported [1] or implant-borne FPDs [2] are the other two treatment options. However, while the former is very destructive to healthy abutment teeth [3], the latter is costly, may cause dental anxiety due to the surgical intervention and yield peri-implantitis when oral hygiene is not optimum [2]. All ceramic resin- bonded IRFPD could be a conservative treatment approach to replace a single posterior missing tooth. However, these types of restorations often failed because of the limited mechanical properties of early generation of dental ceramics [4].

Dental ceramics have evolved significantly during the several decades one of which is zirconia, a high-strength dental ceramic [5]. Zirconia exists in three crystalline states at different temperatures [6]. Partially stabilized zirconia is mainly composed of tetragonal crystals achieved by the addition of 2 to 3% mol Y_2_O_3_ [7]. 3 mol% yttria stabilized tetragonal zirconia polycrystal presents high mechanical properties with superior resistance to fracture and has been increasingly used for frameworks in FPDs in posterior region in the mouth [8,9]. However, since it is quite opaque, it often veneered using feldspathic porcelain through layering technique or using pressable glass-ceramics [5,9]. Nevertheless, chipping of the porcelain veneer is a major complication of these restorations which might be circumvented by the use of full-contoured monolithic translucent zirconia without veneering porcelain [10].

One of the methods of improving the translucency is to increase the yttria content to 5% or more. However, the resultant microstructure consists more of cubic phase which has lower mechanical properties [7,11]. There is little knowledge about the mechanical behavior and reliability of monolithic translucent zirconia used for IRFPDs. Moreover, zirconia is chemically stable [12] and lack of glassy matrix due to its high crystalline content. In fact, adhesion of the resin-based luting cement is essential for the longevity of IRFPDs but high crystalline content of zirconia makes it resistant to conventional conditioning methods used for silica-based ceramic (i.e. hydrofluoric acid (HF) etching and silanization) [12–14]. Different types of mechanical and chemical surface conditioning methods have been recommended to date. Air-abrasion with aluminum oxide particles (Al_2_O_3_) is the most commonly used mechanical treatment [14,15]. Among chemical conditioning methods, Kitayama et al. showed that fusing of a thin layer of silica based ceramic of zirconia ceramic followed by silanization can improve bond strength of resin cement [16]. Another approach leading to chemical interaction with zirconia is the use of functional monomers having an affinity for metal oxides which can be included in the resin cements and adhesives. Phosphate ester monomers, such as 10-methacryoloyloxydecyl dihydrogen phosphate (10-MDP) and phosphoric acid acrylate monomer are among these functional monomers [12,13].

Lithium-disilicate glass-ceramic is another high strength material with impressive esthetic quality [5]. However, its limited mechanical properties may not be promising for posterior FPDs [17]. New microstructure in glass-ceramics has been recently developed with the optimized behavior in mechanical properties and optical features [5]. This novel microstructure is lithium silicate glass-ceramic reinforced with zirconium dioxide crystals [5]. The zirconia reinforced lithium silicate glass-ceramic revealed higher mechanical properties including flexural strength (444 ± 39 MPa), elasticity modulus (70.4 ± 2 MPa) and fracture toughness 2.3 ± 2 MPa m^0.5^ compared with lithium disilicate which presented lower values for the same properties 348 ± 29 MPa, 60.6 ± 1.6 MPa and 2 MPa m^0.5^, respectively [18]. This ceramic can be etched with HF and cemented with adhesive luting materials [19].

This study was designed to evaluate the load at fracture and failure types of 3 units all ceramic FPDs with two different retainer designs, namely full-coverage versus inlay retained FPDs. The ceramic types included either monolithic zirconia that received different types of surface conditioning or a zirconia reinforced lithium silicate glass-ceramic when adopted with a protocol concerning preparation. 3-Unit full-coverage monolithic zirconia FPDs were considered as the control group. The null hypothesis were that neither retainer design, full coverage versus inlay, nor type of material, monolithic zirconia or zirconia reinforced lithium silicate, would affect the fracture load of 3-unit FPDs.

## Material and methods

The materials used in this study along with their batch numbers are summarized in Table 1.

**Table 1.** Materials used in this study (data obtained from the manufacturers).

Brand	Material	Chemical composition	Batch number	Manufacturer
Vita Suprinity	Zirconia-reinforced lithium silicate ceramic	Zirconium-oxide: 8–12% Silicon dioxide: 56–64% Lithium oxide: 15–21% Lanthanum oxide: 0.1%	0000048421	VITA Zahnfabrik, Bad Säckingen, Germany
Ceramill Zolid FX Multilayer	Super-high translucent zirconia	Zirconium-oxide + yttrium oxide + hafnium-oxide: ≥99%. Yttriumoxide: 8.5–9. 5%. Hafnium-oxide ≤.05%. Aluminium-oxide ≤.05%. Other oxides ≤1%	1811001	Ammann Girrbach AG, Koblach, Austria
Panavia F2.0	Primers	Primer A: HEMA, MDP, 5-NMSA, water, accelerator. Primer B: 5-NMSA, accelerator, water, sodium benzene sulfinate	000064	Kurary Co. Ltd, Osaka, Japan
	Resin composite cement	Paste A: MDP, DMA, silanated silica filler, dl-camphorquinone. Paste B: DMA, silanated barium glass filler, sodium fluoride		
Clearfil Ceramic Primer	Ceramic primer	3-MPS, MDP, ethanol	380,024	Kuraray Noritake Dental Inc., Tokoyo, Japan
Initial Zr-FS Feldspar	Silica based ceramic		1707191	GC Europe, Leuven, Belgium

HEMA: 2-hydroxyethyl methacrylate; MDP: 10-methacryoloyloxydecyl dihydrogen phosphate; 5-NMSA: N-methacryloxyl-5-aminosalicylic acid, DMA: dimethacrylates, 3-MPS: 3-methacryloxypropyl trimethoxysilane.

### Experimental model

The experimental models simulating two maxillary abutments (first premolar and first molar) for the replacement of maxillary second premolar (having a span of 6 mm) were used to fabricate full-coverage FPDs or IRFPDs. Sixty-four intact non-caries human maxillary premolars and molars of similar size which were extracted for periodontal or orthodontic reasons were selected and cleaned by scaling and stored in 0.5% chloramine solution [20]. All teeth were visually examined under a microscope (Leica, LEICA EZ4D, MEL SOBEL Microscopes, Italy) and those which were found to be sound and free of cracks or fracture line were used for this study.

In order to homogenize the groups, teeth were selected for the study if the variation in length and width was within 1 mm of the mean values (anatomic premolar crown length = 7.5 mm and anatomic molar crown length = 6 mm; mesiodistal dimension of premolars at the cemento-enamel junction (CEJ) = 5 mm, buccolingual dimension of premolars at the cementoenamel junction (CEJ) = 8 mm, mesiodistal dimension of molars at the CEJ= 8 mm and buccolingual dimension of molars at the CEJ = 9 mm.

Pairs of premolars and molars were randomly assigned into one of the following groups (*n* = 8 per group):

1. Control group (full-coverage monolithic zirconia FPD) (Zr-FPD).
2. IRFPD made of monolithic zirconia (Zr-IRFPD).
3. IRFPD made of monolithic zircona internally coated by a feldspathic porcelain layer (ZrC-IRFPD).
4. IRFPD made of zirconia reinforced lithium silicate, Vita Suprinity (VS-IRFPD).

Prior to the experiments, the roots of the teeth were covered with a thin layer of a light body elastomeric impression material (Panasil initial contact X-light, Kettenbach GmbH & Co. KG, Germany) in order to simulate periodontal ligament membrane. In order to make it comparable to the biological width, the impression material was removed 2 mm short of the CEJ, using a scalpel. Each pair of premolar and molar was embedded in a metal box filled with an auto-polymerizing acrylic resin (Acropars. Marlic Co.) at a distance of 6 mm to simulate a missing second premolar. The abutments were aligned both vertically and horizontally and the occlusal tables of the abutments were set parallel to the horizontal plane.

**Figure 1.**
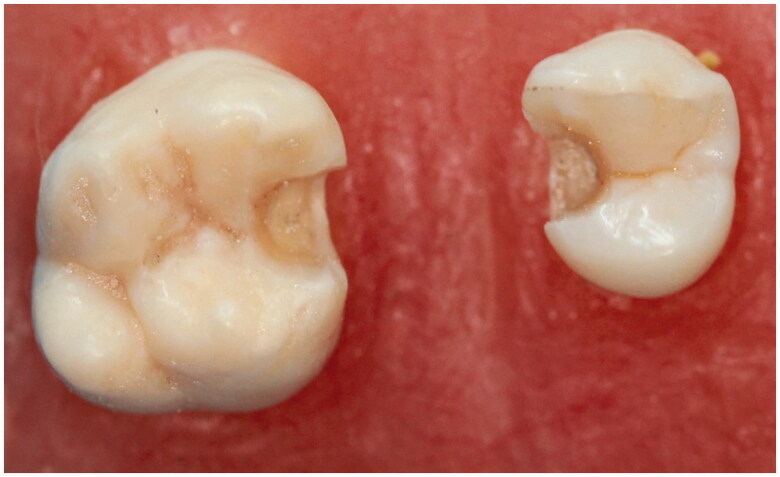
Preparation of the inlay cavities on premolar and molar.

For IRFPD groups, the previously described inlay preparation design [21] was followed to create internal walls with 20° of total convergence angle. A distal inlay cavity with 10° of divergence of each wall and rounded internal angles was prepared with a diamond bur (Meisinger #846-012-FG, USA, LCC) with 3 mm of the intercuspal distance, 2 mm pulpal depth and 4 mm occluso-gingival height. The same procedure was performed for preparing a mesial inlay cavity for a molar. The gingival finish line was a shoulder and no bevels were prepared (Figure 1). Therefore, the dimensions of the connector were 4 mm × 3 mm. A milling machine (IMPLA R 3D-THETA, Schutz Dental Group, Germany) was used to measure the inclination of facial/lingual and axial wall of the inlay preparation relative to the line drawn to the preparation in order to ensure that the preparation angles were 10°. Abutment teeth in the Zr-FPD group were prepared with a 1 mm deep chamfer diamond (Meisinger # 856-012-FG, USA, LCC) with rounded angles circumferentially and 1.5 mm occlusal reduction was performed.

All preparations were made with a high-speed hand piece utilizing water spray coolant by one operator. A new diamond was used after every four preparations.

All preparations were scanned (Ceramill Map 400+, Amann Girrbach AG, Austria) and a virtual spacer layer of 40 µm were chosen. Eight full-coverage zirconia bridges and 16 inlay-retained zirconia bridges were manufactured by a CAD/CAM system (Ceramill motion 2, DNA, Ammann Girrbach AG, Austria) using partially sintered monolithic zirconia (Zolid fx multilayer, Ammann Girrbach AG, Koblach, Austria). After the milling procedures, the enlarged restorations were removed from the CAD machine and final sintering was performed in a special furnace at 1450 °C for two hours. For the ZrC-IRFPD group of the study, the same procedure was followed but 30 µm additional space layer was chosen in CAD system for internal coating of zirconia IRFPDs by a silica-based ceramic (Initial Zr-FS feldspar, GC Europe, Leuven, Belgium) with a matching coefficient of thermal expansion. A layer of a separating medium (Vita Modisol, VITA Zahnfabrik, Bad Säckingen, Germany) was applied on the die before the porcelain mixture was added into the intaglio surfaces of the retainers of the fabricated zirconia IRFPDs. The separating medium facilitated removal of zirconia IRFPDs without any distortion of the applied porcelain prior to firing. ZrC-IRFPD was then seated on the abutments with gentle finger pressure. Extra amount of porcelain extruded from the cavity margins were removed by a brush. The porcelain was then fired at 810 °C for 1 min under vacuum.

The VS-IRFPDs with the required dimensions were cut out from the respective blocks (Vita Superinity PC) using a milling machine (InLab MC XL, Sirona, Germany) which was followed by an additional thermal cycle in the furnace (Vita Vacumat 6000 MP, Vita Zahnfabrik, Bad Säckingen, Germany) according to the manufacturer`s instruction.

The prepared prostheses were finally cleaned with steam and then seated on the abutment teeth. While constant finger pressure was applied onto the FPDs, they were evaluated on the abutment teeth by visual inspection under a microscope (Leica, LEICA EZ4D, MEL SOBEL Microscopes Ltd., Italy) at a magnification of 8× for marginal discrepancy. The inspection was performed on the buccal, lingual and mesial aspects of premolar abutments and the buccal, lingual and distal aspects of the molar abutments for the Zr-FPDs. For IRFPDs, the gaps were measured at the facial and lingual interproximal margins and along the occlusal margins. The restorations were rejected if the marginal discrepancy was greater than 60 µm according to the literature [22]. New FPDs were fabricated on the same abutment teeth to replace the rejected specimens.

### Luting procedure

A dual-polymerized resin cement (Panavia F 2.0) was used for cementation of all groups according to the manufacturer`s instruction.

For groups Zr-FPD and Zr-IRFPD, the surfaces were air-abraded (50 µm Al_2_O_3_) for 20 s at 2 bar pressure from a distance of 10 mm. In order to ensure that the air-abraded surfaces were free from loose alumina particles, the FPDs were cleaned with steam. The internal surfaces of ZrC-IRFPD and VS-IRFPD were etched with 9.5% hydrofluoric acid gel (Bisco, Inc., Schaumburg, IL) for 1 min and 20 s, respectively. They were then washed thoroughly and dried.

After these treatment procedures, the surfaces of all groups were conditioned with a primer (Clearfil Ceramic Primer, Kuraray Noritake Dental Inc., Tokyo, Japan) and gently air-dried.

**Figure 2.**
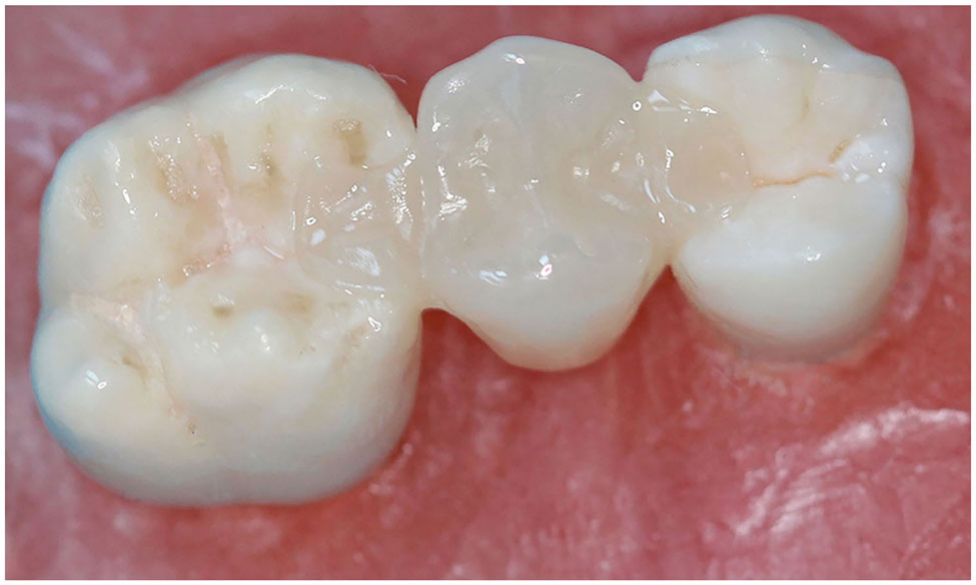
Test specimen for inlay retained fixed partial denture.

All abutment teeth were etched with 35% phosphoric acid (K-Etchant Gel, Kuraray Noritake Dental Inc., Tokyo, Japan) for 15 s, rinsed and blot-dried prior to cementation procedure. Afterwards, equal amounts of ED PRIMER A and B were mixed and applied to the abutment surfaces and dispersed with an air syringe. Then, equal amount of paste A and B of Panavia F 2.0 cement were dispensed and mixed and applied on the inner surface of all retainers. The FPDs were placed on the abutments and held in place with finger pressure for 40 s. Any excess cement was removed with a micro-brush and glycerin gel was applied in the marginal areas (Figure 2). The margins in all surfaces were photo-polymerized for 20 s (Bluephase, Ivoclar Vivadent, Schaan, Liechtenstein). All cementation procedures were performed by the same operator and a dental assistant.

All specimens in each group were then subjected to thermo-cycling (5000 × 5–55 °C) with 30 s dwell time before the testing procedures. The FPDs were then centrally loaded in a universal testing machine (Zwick/Roell, ProLine Z050, Berlin, Germany) at a cross-head speed of 0.75 mm/min with a stainless steel ball (diameter: 5 mm) placed on the center of the pontic. To achieve an even force distribution, a 0.5 mm tin foil was placed between the pontic and the loading ball. The fracture loads were determined when a sudden decrease in the applied load occurred. Values of fracture loads (N) were recorded and modes of failure were determined with a stereomicroscope (Leica EZ4D, Leica Microsystems Ltd., Heerbrugg, Switzerland). The nature of the fracture patterns was classified as following: Cohesive fracture in ceramic, pontic or connector area and adhesive failure at the ceramic-resin interface.

### Statistical analysis

Normal distribution of data was tested using one sample Kolmogorov-Smirnov test (SPSS 20 statistical package, SPSS Inc., Chicago, IL, USA). The data were then analyzed using one-way analysis of variance (ANOVA) and multiple comparisons were performed with the post-hoc test (Tukey-HSD). *p* Values less than .05 were considered significant in all tests.

## Results

Mean and standard deviations of the fracture load values are presented in Table 2. There was statistically significant difference among the groups (*p ˂* .001). The values for VS-IRFPD (391 ± 136 N) were significantly lower than those of all the other groups (Zr-FPD, Zr-IRFPD, and ZrC-IRFPD) (*p* ≤ .025). The mean failure load for Zr-FPD group (672 ± 183 N) was not statistically different from those of Zr-IRFPD (672 ± 123 N) and ZrC-IRFPD (638 ± 59 N) (*p* ≥ .998).

**Table 2.** Failure load (N) for four test groups (*n* = 8 per group).

Groups	Mean	SD	Min	Max
Zr-FPD	672^a^	183	453.04	931.35
Zr-IRFPD	672^a^	123	480.61	811.25
ZrC-IRFPD	638^a^	59	638.63	734.16
VS-IRFPD	391^b^	136	204.02	587.20

Identical superscript letters indicate no significant difference (*p* ≥ .05). Zr-FPD, complete coverage zirconia fixed partial dentures; Zr-IRFPD, zirconia inlay retained fixed partial denture (air-abraded); ZrC-IRFPD, inlay retained bridge from zircona internally coated by a feldspathic porcelain layer; VS-IRFPD, zirconia reinforced lithium silicate inlay retained fixed partial denture.

**Figure 3.**
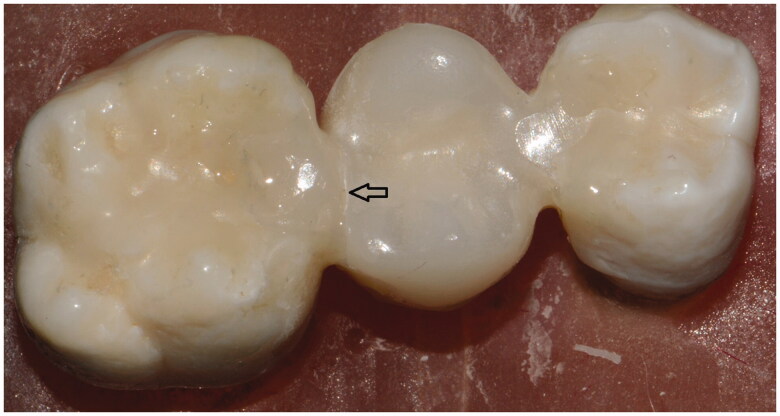
Ceramill Zolid FX Multilayer IRFPD where failure occurred in connector.

**Figure 4.**
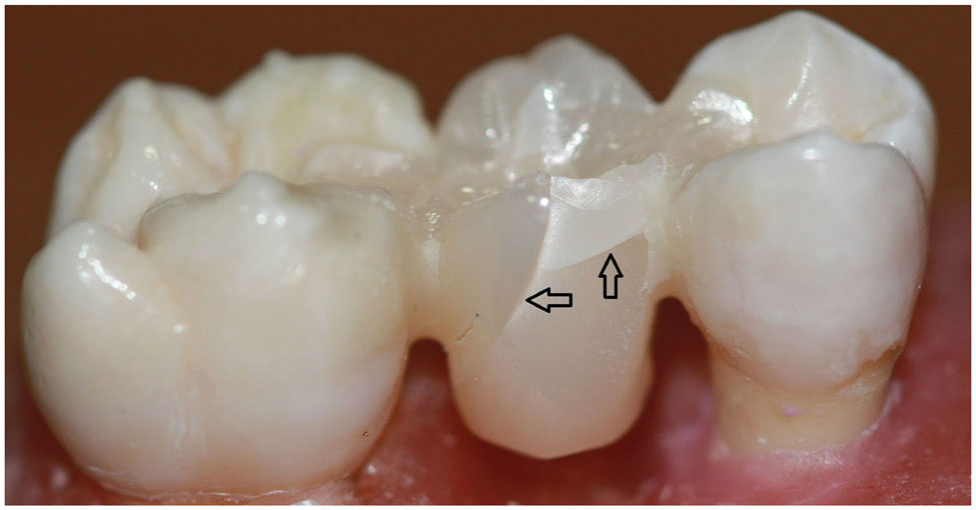
Ceramill Zolid FX Multilayer IRFPD where failure occurred in pontic.

**Figure 5.**
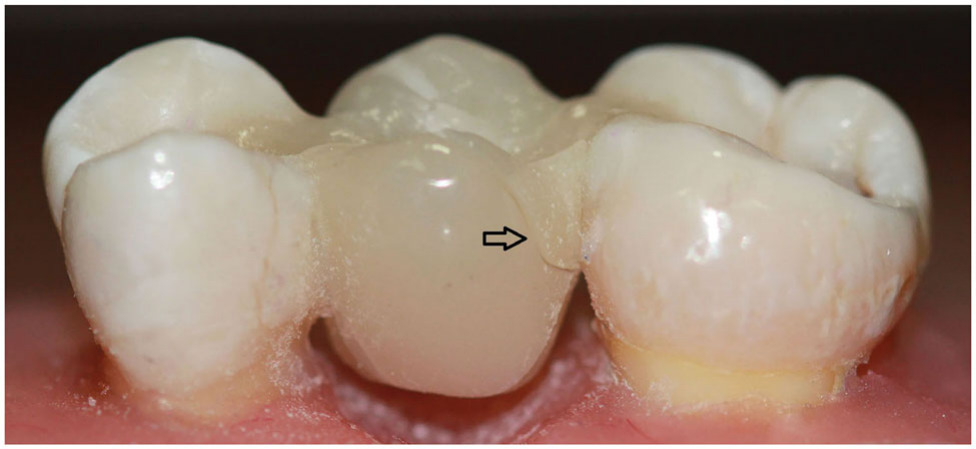
Vita Suprinity IRFPD where failure occurred in pontic.

All groups failed cohesively and no adhesive failures were observed. The failure sites are illustrated on Figures 3–5. In Zr-FPD group, failures were one in pontic and seven in connector region. The fracture patterns of IRFPDs made from zirconia were similar (three in pontic and five in connector). Specimens in VS-IRFPD displayed equal numbers of cohesive failures in both connectors and pontic.

## Discussion

In this study, the fracture load of 3-unit inlay-retained fixed partial dentures made from either monolithic zirconia or zirconia reinforced lithium silicate were tested and compared with that of 3-unit full-coverage fixed partial dentures made from monolithic zirconia. The inlay-retained monolithic zirconia FPDs received two types of surface treatments. All FPDs were designed to restore maxillary second premolar. Inlay-retained monolithic zirconia FPDs showed fracture load which were not statistically different from 3-unit full-coverage monolithic zirconia FPDs, regardless of type of surface treatment. Inlay-retained FPDs made from zirconia reinforced lithium silicate failed at a significantly lower load than the other types of FPDs. Thus, the first null hypothesis about the non-significant effect of retainer design on fracture load was accepted. However, the second null hypothesis that ceramic type is inconsequential on fracture load was rejected.

In agreement with these results, several previous studies showed that zirconia inlay-retained FPDs exhibited higher resistance to fracture when compared to lithium disilicate inlay-retained FPDs [23,24]. The difference may be related to their varying level of mechanical properties. Flexural strength of 444 MPa, elastic modulus of 70 GPa and fracture toughness of 2.3 MPa m ½ have been reported for Vita Suprinity [23,24]. The result of a study made by Elsaka, showed fracture toughness value of 3.7 MPa m½ and flexural strength of 676 MPa for Ceramill Zolid FX Multilayer [25]. In an in vitro study, the elastic behavior of CAD/CAM materials were compared and polycrystalline zirconia demonstrated Young’s modulus and bulk modulus of 206 GPa and 134 GPa, respectively which were almost twice the values demonstrated by zirconia reinforced lithium silicate [26].

The average load bearing capacity of 3-unit full-coverage and inlay-retained FPDs recorded in this study exhibited mean values ranging between 391 and 672 N. Numerous authors have investigated the maximum bite forces during mastication in different region [27] and have reported a different range from 200 N in anterior region to 350 N for posterior area [28]. The highest bite force, however, has been reported in the first molar region to be about 500 N [29]. It seems that it is insufficient to rely on the current in vitro examination to predict clinical performance of the ceramic materials for replacement of first premolar in inlay retained FPDs.

In late 1990s, Kelly recommended four factors for in vitro examination of load to failure test of all ceramic restorations to simulate clinical situation: (1) die material with elasticity similar to dentin (2) avoiding point contact but having contact dimensions of 0.5 to 3 mm, (3) cyclic loading, and (4) testing in wet conditions. At the current study, 2 out of 4 the requirements were fulfilled. Another point fulfilled in the current study is the minimum span length required in uniaxial flexural strength test. Minimum length of 20 mm has been allowed in ASTM C 1161, ISO 17565 and ENV 843-1 for ceramics [30]. In the current study, the similar span length was observed considering the mesio-distal dimension of premolar and molar teeth.

The inlay-retained FPDs were subjected to a thermo-cycling process at the present study. It has been demonstrated that aging accelerate degradation of adhesive interface [31]. However, thermal variation did not cause debonding at ceramic-resin interface during 5000 thermocycles in all groups in the current study. In addition, different surface conditioning of zirconia had no effect on fracture load implying that adhesive interface was resistant to hydrolytic degradation in this short period of thermal aging process. Nevertheless, long lasting adhesion of resin cement to zirconia surface internally coated by fusing silica-based ceramic is not clear and requires further studies. In addition, forces are not always applied perpendicular to the axis of a restoration as in the current in vitro study and it is more clinically relevant to test the FPDs under fatigue load [17,32,33]. As a result, translation of the results of this in vitro study to the reality of the oral cavity is limited. Yet, some ranking could be made between materials before clinical trials are contemplated.

Failure modes at full-coverage and inlay retained FPDs were fractures in ceramic either in connector or pontic, being predominantly at connector area. No de-bonding was observed in any of the specimens. It might indicate a favorable surface conditioning of monolithic zirconia through feldspathic layering and/or air-abrasion. In addition, use of an MDP-containing resin cement might have resulted in a stable adhesive interface during 5000 thermocycling and static loading. Furthermore, two methods of surface conditioning of monolithic zirconia inlay-retained FPDs were comparable regarding load bearing capacity. The findings could indicate the high importance of bonding for the success of inlay-retained ceramic FPDs considering the reduced surface area for bonding.

Several studies have demonstrated that the connector areas are highly influential in failure and failure rate is relatively high in 3-unit all-ceramic FPDs associated with the connector area [17,24,29]. Brittle materials, such as dental ceramics are weak in tension [34]. Finite element analysis showed that tensile stress concentrates at the gingival embrasure, and the cervical area of connectors and pontic of ceramic bridges in flexural compressive loading [35–37]. Accordingly, some modifications have been recommended to minimize stress at inlay-retained ceramic FPDs. Increasing the ceramic thickness especially in the connector areas and selecting a ceramic material with a high modulus of elasticity are methods of improving the load bearing capacity of inlay-retained FPDs [21,24,29].

The ideal preparation design for ceramic inlay-retained FPDs described by Thompson et al. [21] was followed in the current study to provide a balance between tooth preparation with minimal effect on tooth strength and adequate bulk in ceramic material [21]. The recommended connector dimensions in all-ceramic posterior inlay-retained fixed partial dentures varied between 9 mm^2^ to 16 mm^2^ [24,28]. Occluso-gingival height of 4 mm has been suggested to reduce the failure probability [17,28]. Thompson et al. also expressed the need to increase the degree of taper from 6 to 8° recommended for cast metal restoration to 20° for ceramic inlay retainer to avoid binding of the restoration during try-in and the likelihood of stress build up [21]. Assessment of tooth preparation for ceramic crowns and onlays in clinical practice demonstrated that internal tapers or convergence angles were frequently greater than 20°, leading to wide occlusal isthmus widths [38]. However, Esquivel-Upshaw et al. reported that degree of preparation tapers inversely influenced the fracture resistance of all-ceramic inlays and the restorations where 5° taper were more fracture resistant than those with 15 and 20° taper [39]. High cohesive failure in the connector area in the current study might be to attributed to the preparation taper of the abutment teeth for the inlay-retainers.

A systematic review on all-ceramic inlay-retained FPDs for replacing posterior missing teeth was conducted in 2018 [24]. The inlay cavity preparation in the included studies followed the preparation design described by Thompson et al. [21] as in the current study. According to this systematic review, zirconia-based inlay retained FPDs could be a treatment option for restoring posterior single missing teeth. However, there was a lack of standardization about the surface conditioning and luting procedures. In spite of all these controversies, Kern in his literature review revealed that air-abrasion with 50 µm alumina particles at 2.5 bars pressure followed by MDP primers and MDP-containing luting resins can provide a long-term clinical durable bonding to zirconia surface in oral environment under humidity and stressful conditions [15]. In another study by Castillo-Oyagüe et al. [24], it was reported that connector is the weakest part of inlay-retained FPDs and it was attributed to the high stress concentration at the junction of occlusal and proximal surface of the inlay retainers [24].

In the present study, fracture load of two types of ceramics in 3-unit FPDs were tested to determine the more suitable material for replacement of single posterior missing tooth. Among the materials tested, monolithic zirconia-based inlay-retained FPDs showed promising results, yielding fracture loads comparable to the fracture loads of full-coverage monolithic zirconia FPDs. The similarity in the fracture load of ceramic FPDs could be attributed to the material properties and connector dimensions [24,40]. In addition, all fracture load values of inlay-retained FPDs obtained in this study were in the average of the assumed maximum mastication forces in the premolar region [27,41]. However, there are some limitations in the current study in terms of clinical relevance, especially regarding mechanical and fatigue loading and therefore further in vitro investigations with higher number of thermal cycles and mechanical loading in an artificial oral environment are required in order to achieve information for long term clinical performance of such restorations.

## Conclusions

Based on the findings of this study, for replacement of second premolar tooth, there was no significant difference in fracture load of inlay-retained and full-coverage fixed partial dentures made of monolithic zirconia under static loading when a connector size of 12 mm^2^ was provided. In contrast, inlay-retained zirconia reinforced lithium silicate fixed partial dentures showed significantly lower load bearing capacity. All failures were cohesive in nature in ceramic materials and the connectors were the weakest parts of the reconstructions.
